# What can simulation educators learn from the reluctant participant? An exploration of the factors influencing engagement amongst adult learners participating in paediatric simulation training

**DOI:** 10.1186/s41077-025-00331-9

**Published:** 2025-02-13

**Authors:** Laura Newhouse, Ngaire Polwart

**Affiliations:** Douglas Starship Simulation Programme, Starship Children’s Hospital, 2 Park Road, Grafton, Auckland, 1023 New Zealand

**Keywords:** Simulation, Medical education, Learner engagement, Psychological safety, Nursing

## Abstract

**Background:**

Simulation educators are typically passionate advocates for simulation as a training modality; however, we frequently encounter participants who do not share our enthusiasm. The voice of the highly engaged participant is well publicised; however, the experience of those who do not readily engage in simulation has not been extensively studied and may offer valuable insights for educators. This qualitative study will explore factors which influence learner engagement in paediatric simulation training, informing the practice and approach of simulation educators to optimise learning experiences.

**Methods:**

We conducted a reflexive thematic analysis of 12 semi-structured interviews with medical and nursing professionals from a large paediatric teaching hospital in New Zealand who self-identified as reluctant participants in simulation-based education. Interviews explored factors which have influenced their engagement in simulation-based education over the course of their careers.

**Results:**

Three overarching themes were developed which describe the factors influencing adult-learner engagement in simulation-based education. The first, participant anxiety, explores the participants’ narratives related to anxiety before, during and even following simulation which can impact on their ability to engage in current and subsequent simulation-based education. The second, protective behaviours, relates to the defensive mechanisms employed by participants in response to vulnerability experienced during simulation activities. The third theme, perception of the facilitator, examines the impact of simulation facilitator characteristics and behaviours on learner engagement.

**Conclusions:**

These narratives highlighted that regular simulation activities with transparent learning objectives in which facilitators demonstrate vulnerability and adopt a co-learner attitude act to reduce participant anxiety. Emergent defensive behaviours, particularly “group shielding”, interfere with collective learner engagement and should be both recognised and addressed by facilitators. Finally, there are potential discrepancies in the perceptions of facilitators and learners regarding what constitutes psychologically safe education environments. A collaborative and iterative approach to simulation-based education design may act to improve psychological safety for reluctant participants.

## Background

Learner engagement is complex, affected by a multitude of emotional, social and situational factors. In any learning setting, it is recognised that learner engagement is key to maximising educational value [1]. Studies have demonstrated that higher levels of engagement amongst learners correlate to higher levels of satisfaction and achievement [1, 2].

Within healthcare, engagement with simulation-based education (SBE) has been shown to be influenced by fidelity, perceived psychological safety, facilitator behaviour and professional seniority of learners [3–5]. Facilitators of healthcare simulation promote engagement through the development of high fidelity and psychologically safe learning environments. While engagement has been favourably associated with these endeavours [3, 6], evidence is limited by the challenging nature of defining and measuring learner engagement [1].

When referring to engagement in this study, we adopted the definition proposed by Padgett et al. [1] which states:

“*Learner engagement is a context-dependent state of dedicated focus towards a task wherein the learner is involved cognitively, behaviourally and emotionally*” (p 819).

Within this definition, engagement is referred to as a state, rather than an inherent trait, whereby a person may be engaged in one context but not another. A challenge for simulation educators is that it is not always obvious when participants are reluctant to engage in learning. Potential cues suggestive of engagement may actually be indicative of false engagement [7]. Interestingly, while active engagement can drive group engagement, it can also have an adverse effect of the engagement of others in the group, for example, when a debrief is dominated by a few individuals [7]. There is a paucity of research investigating contexts in which learners do not feel engaged in SBE. Exploring the socio-emotional aspects of learner reluctance to engage in SBE may provide valuable insight for educators to identify variables that can, or conversely, cannot be changed [8, 9].

The objective of this study is to explore factors which influence learner engagement amongst participants who have self-identified as reluctant to participate in paediatric SBE. When using the term “reluctant participant”, we are referring to those who partake in the simulation activities with hesitation, unwillingness or lack of enthusiasm. They may participate as they feel obligated, pressured or out of a sense of duty, rather than out of genuine interest or eagerness. We will achieve our study objectives through reflexive thematic analysis of semi-structured interviews with simulation participants in a paediatric teaching hospital in New Zealand. The findings will inform the practice and approach of simulation educators to optimise participant engagement and learning experience.

## Methodology

### Study context

The study was undertaken at a tertiary paediatric hospital in New Zealand with a well-embedded simulation programme.

### Research team and reflexivity

The authors brought a variety of knowledge and experience to the study. LN is a paediatric doctor working as a simulation fellow with extensive experience of both participating in and facilitating simulation-based education. NP is a psychology masters graduate with experience in healthcare research, working in an administrative role within medical research and education at the time of the study.

The subjectivity of the researcher is integral to reflexive thematic analysis, and reflection on one’s own assumptions and prior training is crucial to analysis. This method facilitated the key research outcome of developing strategies to address modifiable factors within teaching design and delivery. A reflective approach fostered growth and development of the researchers as the data was analysed.

### Recruitment, sampling and eligibility

Between February and June 2024, 12 medical and nursing professionals were recruited from those who participate in the established simulation programme at Starship Hospital in Auckland, New Zealand. Purposive sampling was employed to enrol those who self-identified as experiencing current or previous reluctance to participate in SBE. Students and other temporary visitors to the clinical setting were excluded from the study.

The study was explained to eligible individuals at the time of participating in SBE within the hospital. Thirty-three eligible professionals expressed an interest in participating and provided their email address to be contacted with further information. A participant information form was emailed to prospective participants with an invitation to schedule an interview. Follow-up emails were sent if there was no response within 1 month of initial email correspondence. Prior to the interview, participants had an opportunity to ask questions about the study and formal written consent was obtained. Twelve participants agreed to take part in an in-person or online interview at a convenient time. Of the 21 eligible individuals who did not participate, 19 did not respond to follow up emails and 2 were unable to schedule a convenient time for interview. Of the 12 enrolled participants, there were 6 doctors and 6 nurses. Ten of the participants were female and 2 were male. Participants had between 6 months and 19 years post-graduate professional experience.

### Data collection

Enrolled participants took part in a semi-structured interview conducted by NP for a duration of 20–40 min. The format of semi-structured interviews was chosen as it allowed for an informal guided process to gather retrospective accounts of participants’ experiences in simulation and further in-depth probing into the socio-emotional aspects of their responses. The full semi-structured interview guide is available as supplementary material and example questions are provided in Table 1; however, the interview was very much a fluid process with questions arising from information that individual interviewees provided. To minimise courtesy bias, NP conducted the interviews as NP does not facilitate SBE sessions or work directly with any of the participants.

**Table 1:** Sample interview questions

What feelings do you attribute to your early experiences in simulation?
Have your feelings toward simulation changed over the course of your career?
Could you describe how you feel when participating in simulations with people you are familiar with (i.e. work with every day) as opposed to doing simulations with people who you are not so familiar with?
What qualities do you think make a good simulation facilitator?
What team or facilitator factors do you think lead to a less successful simulation?
Do you think the facilitators could modify anything to help participants feel more engaged in simulation?

Interviews took place either face-to-face or via Microsoft Teams depending on participant preference. With permission, all interviews were live transcribed using Microsoft Teams. All participants were asked if they would like to review their transcripts prior to analysis. Those who wanted to see transcripts were given 2 weeks to provide any corrections. Of those reviewing transcripts, no amendments were required. Recordings and transcriptions were securely stored in a password protected hospital database. The interview transcripts were manually reviewed for accuracy against the audio recordings and transcriptions were de-identified prior to analysis. A data management plan was completed regarding the storage of data from this study in accordance with local information governance policy.

### Data analysis

We used a reflexive thematic analysis research methodology, employing the six phases of thematic analysis as described by Braun and Clarke [10]. This method was chosen as it provided a systematic approach to increase understanding of participants’ experiences, ideas and perceptions as well as capturing patterns of meaning across the dataset related to central theme of participation reluctance. A critical realist analytical framework as described by Fryer [11] was employed using an inductive, data driven approach to identify causal relationships between experiences, beliefs and behaviours.

Familiarisation with the dataset was achieved through repeated reading of the transcripts. Initially, the data was coded in sections and subsequently line by line by LN. During this process, ideas and meanings were noted to capture initial impressions. Following development of principle codes, a visual mind-map was created to draw connections between codes and generate initial themes. Themes were reviewed throughout the analytic process by both LN and NP. This iterative process aided deeper understanding of the data and led to extensive re-coding and mapping until final themes were agreed.

### Ethics

Ethics approval was granted by Auckland Health Research Ethics Committee (AHREC) on the 18th of December 2023 (Reference: AH26421).

## Results

Three overarching themes are presented from reflexive thematic analysis which describe the factors influencing adult-learner engagement in simulation-based education: (1) participant anxiety; (2) protective behaviours; and (3) perception of facilitator. An overview of main themes and subthemes is provided in Table 2.

**Table 2:** Selected quotes

Theme 1: Participant Anxiety	
Anticipation	*“I could feel my anxiety going up and up and then when I actually got there, I was just paralysed” Participant 10* *“I was that stressed that like, I needed to get out – but I couldn’t get out, so I had to go through” Participant 5* *“if you have tachycardia going into a sim then I think that’s pretty normal!” Participant 10* *“You don’t really know what’s going to happen, that sort of thing, knowing what’s going—what we’re going to face” Participant 6* *“it comes down to that fear, the fear of not knowing. The fear of doing something wrong. Probably the fear of being judged…yeah you know the worst things” Participant 9* *“I could feel my heart racing” Participant 4* *“I get sweaty and nauseated” Participant 1*
Reputational ramifications	*“There’s no way that can’t impact on someone’s impression of you, do you know what I mean?” Participant 8* *“I didn’t want to look bad in front of other people who might come across my name in the future” Participant 10* *“A lot of the big bosses were [running the sim]” Participant 10* *“you don’t want to disappoint…you don’t want them to think you didn’t know how to do this procedure or how to manage this emergency” Participant 1* *“the fall out afterwards thinking you haven’t done a good job” Participant 10* *“you sort of start to question your ability and your own skill I guess” Participant 9*
Under scrutiny	*“it’s a performance” Participant 3* *“I’m not comfortable when someone is watching” Participant 7* *“[being watched] hinders a proper execution of your job” Participant 7* *“I think people perform a bit differently when they know they’re being filmed. I think people don’t perform naturally and which is a bit of a laugh actually” Participant 3* *“With like the video recording you get to see how you were doing and how everybody was doing…you can see like an overview” Participant 2* *“That could be what I’m doing during real resus” Participant 2*
Anxiety as inevitable	*“I think that anxiety is kind of part of the point. Like that’s kind of the purpose of it” Participant 8* *“they are meant to do that—to pressure us…they’re just doing their job” Participant 7*
Theme 2: Protective Behaviours	
Defensiveness	*““Suddenly everyone got their backs up…now I’m just gonna armour up and perform well myself” Participant 6* *“I’m always a little bit standoffish, but yeah, I think it just goes back to that fear” Participant 11* *“I started blaming the simulation and then the whole thing goes…” Participant 6*
Group shielding	*“It was kind of personal…just focussing on a mistake that person has done…we are also team members…this is a team effort not a one man show.” Participant 2* *“There’s no way I’m bringing my paediatric registrars into a sim situation…because previously they have been so traumatised” Participant 10*
Avoidance	*“I was purposefully avoiding it due to previous trauma with sim and that’s also why I was facilitating the study days so I wasn’t on the receiving end…it was organised by me for that reason.” Participant 10* *“I’m such a hypocrite because I’m like “away you go”[to the junior nurses]” Participant 9* *“it was just so appalling and we’re not coming back” Participant 10*
Theme 3: Perception of the Facilitator	
Practice what you preach	*“ I feel some [educators] say [the pre-brief] but they don’t come across as genuine…or they then go and talk about their performance behind their back or something. So then it’s basically like a lie.” Participant 6* *“I don’t think it just breaks down the trust for that session…it sets the culture. So I think you have to set it from the beginning and be consistent” Participant 6* *“if someone uses [that word] on me I just shut down” Participant 10* *“if it’s going to be advertised as this learning – this psychological safe environment – it needs to – that’s what it needs to do” Participant 6*
Build trust	*“a bit of a spiel” Participant 6* *“they always say, you know, none of this goes beyond this room and all that sort of stuff” Participant 1* *“it’s always stressed it’s a safe environment etcetera etcetera. But I don’t know, you still worry about it.” Participant 12*
Communicate vulnerability	*“…they* [the facilitators] *recognised that they were also learning. So they were learning how to be facilitators and so now they have grown. So that was really helpful for me to understand that.” Participant 10* *“[it was an] absolute change… like a moment in time changed my life…this has actually happened to others—to multiple people—and now they’re trying to work to change that. It’s like I was validated.” Participant 10*
Be open and curious	*“If that can be explained to you at the start, you know, this is all about being open and curious” Participant 10* *“…whereas reflecting back, if they’d* [the facilitators] *said to me “what was going on?”… I would have been able to be more honest.” Participant 6*

### Participant Anxiety

Many participants described feelings of intense anxiety in relation to their simulation experiences, which impacted on their ability to engage. On deeper exploration, the drivers of anxiety were multifaceted and interwoven.

Anticipatory anxiety in advance of simulation was described repeatedly and compounded by a sense of obligation to attend and participate in teaching. Participants frequently used language of endurance to describe the teaching sessions, comparing it to getting through an ordeal or being trapped.

*“I was that stressed that like, I needed to get out – but I couldn’t get out, so I had to go through” - Participant 5*

*“I could feel my anxiety going up and up and then when I actually got there, I was just paralysed” - Participant 10*

Uncertainty about the content of the teaching led to a reluctance to attend teaching and this was inextricably linked to a fear of appearing to lack knowledge or skill in front of colleagues.

*“I didn’t want to look bad in front of other people who might come across my name in the future” - Participant 10*

Concern about judgement and reputational damage on the basis of performance in simulation was a recurrent feature across transcripts and was particularly acute in the context of a hierarchical relationship. Power imbalance, both between members of participant group as well as between facilitator and participant, was professed as inevitable but also recognised to have a negative impact on open and honest learning conversations. There was a perception that poor performance in simulation could have a long-lasting impact on career and professional standing. This idea was pervasive when participating in simulation in both native and non-native teams.

*“There’s no way that can’t impact on someone’s impression of you, do you know what I mean?” - Participant 8*

The fear of making mistakes publicly in simulation led one participant to question their professional identity and ability. A sense that poor performance would not only affect their reputation but also undermine their sense of self-worth and professional pride.

*“…you sort of start to question your ability and your own skill I guess” - Participant 9*

The practice of being observed during simulation had very negative connotations and references to performance and acting were commonly made. This performance anxiety was described as altering usual behaviours and negatively impacting execution of clinical tasks. Paradoxically, the use of video recording in debriefing was described as positive by those who had experienced it. The overview of team performance was highly valued as was the opportunity to review real-time action and reflect on practice. Although acknowledged that watching the video back as a group was awkward on occasion, on balance, it was felt that the educational benefits outweighed the social discomfort.

*“With like the video recording you get to see how you were doing and how everybody was doing…you can see like an overview” - Participant 2*

*“I think people perform a bit differently when they know they’re being filmed. I think people don’t perform naturally and which is a bit of a laugh actually” - Participant 3*

It was felt by many that anxiety is an inevitable part of simulation. Some perceived that the goal of simulation was to evoke anxiety in participants in order to maximise fidelity and immersion. The role of the facilitator in creating a highly pressurised simulation experience was seen by some as integral to their job.

“I think that anxiety is kind of part of the point. Like that’s kind of the purpose of it” - Participant 8

Many acknowledged the benefits of emulating the high stress clinical environment of real life whilst in the confines of a simulated setting; however, the pressure was also perceived to negatively impact participant engagement and lead to simulation avoidance behaviours.

### Protective Behaviours

This theme explored the individual and team protective behaviours which participants described in response to feelings of vulnerability evoked during SBE.

Defensiveness was a common behaviour described particularly when feeling singled-out during the debriefing conversation. Comments on the limitations of fidelity or set-up of the simulation were attributed defensive mechanisms and were detrimental to the educational value of the teaching. Furthermore, self-defensive attitudes resulted in an impulse to prove oneself in terms of skill or competency, leading to a loss of focus on teamwork. This is likely to limit attainment of group learning objectives.

*“I’m just gonna armour up and perform well myself” - Participant 6*

Team-protective behaviours were invoked as participants described gathering around a group member who they perceived to be isolated in the debrief. We have termed this phenomenon “group shielding”. We propose that this behaviour either arises from or leads to an “us and them” mind-set and creates division between facilitator and participants. This may subsequently affect the ability of the facilitator to engage participants in a productive debriefing conversation.

*“Suddenly everyone got their backs up” - Participant 6*

*“It was kind of personal…just focussing on a mistake that person has done…we are also team members…this is a team effort not a one man show.” - Participant 2*

The narratives of some participants described complete avoidance of simulation in response to previous traumatic simulation experiences. We ascribed this behaviour to an extreme mechanism of self-defence. Notably, some had taken leadership roles within medical education allowing them to facilitate rather than participate. This level of disengagement inevitably limits educational opportunities.

*“I was purposefully avoiding it due to previous trauma with sim and that’s also why I was facilitating the study days so I wasn’t on the receiving end…it was organised by me for that reason.” - Participant 10.*

### Perception of facilitator

We ascertained that the characteristics and behaviours of the facilitator were of crucial significance to the participants and were intrinsically linked to learner engagement. Participants highlighted the importance of the facilitator being considerate in the language they use, and their behaviour being grounded in authenticity.

*“I feel some people [educators] say [the pre-brief] but they don’t come across as genuine…or they then go and talk about their performance behind their back or something. So then it’s basically like a lie.” - Participant 6.*

The integrity of the facilitator was highly valued and was perceived as key in creating a psychologically safe learning environment founded on trust. Participants felt strongly about the need for facilitators to act and speak with honesty and to uphold the principles of The Basic Assumption (see glossary) and confidentiality. The breakdown in trust when this did not occur was not only felt to be damaging to engagement in that particular teaching session but to have longer lasting impact on future participation.

*“I don’t think it just breaks down the trust for that session…it sets the culture. So I think you have to set it from the beginning and be consistent”* - *Participant 6*

*“There’s no way I’m bringing my [juniors] into a sim situation…because previously they have been so traumatised” - Participant 10*

The language used by facilitators was seen as significant in promoting honest dialogue. Direct feedback was seen by some as beneficial for learning and more memorable whereas others described that comments on personal performance precluded effective learning conversations. The power of language was highlighted by one participant who stated that the use of certain single words during the debrief had in her experience led to a complete disengagement in conversation.

“*If someone uses [that word] on me I just shut down”* - *Participant 10*

Simulation theory emphasises the use of pre-simulation activities in establishing a psychologically safe learning environment which is fundamental to learner engagement. A number of participants described the pre-brief using quite glib terminology, such as “a bit of a spiel”, conveying a lack of faith in the sentiment of these pre-simulation statements.

*“It’s always been stressed it’s a safe environment etcetera etcetera—but I don’t know, you still worry about it”* - *Participant 2*

An open and curious approach on the part of the facilitator was stressed as a very positive trait, as was an acknowledgement of past simulation experience and the impact that may have on participant emotions and approach to the learning environment. To voice the notion that we are all learning, both in the role of participant and facilitator were felt to both lessen the hierarchy and to foster a more nurturing educational environment.

## Discussion

The narratives from our study gave voice to an under-represented group within SBE literature. Their perspectives should steer the design and practice of SBE to optimise participant engagement and learning experiences. Three main themes were generated from the data that were suggestive of influencing adult learners’ hesitancy to engage in SBE: (i) participant anxiety; (ii) protective behaviours; and (iii) perception of the facilitator.

Anxiety and fear were dominant emotional responses described by participants when discussing their simulation experiences. The relationship between these stress responses and learning engagement is complex. The impact on cognition, emotion and behaviour may be beneficial or detrimental and is dependent on the individual as well as the intensity of the emotion and chronicity of exposure to stressful stimuli [12–14]. Some participants in our study acknowledged that a certain level of anxiety was necessary in simulations to realistically reflect emotions experienced during clinical events. Indeed, research has shown that a certain level of stress during SBE is advantageous in preparing healthcare professionals to work in similar stressful real-world environments [9]. While a certain degree of pressure can have beneficial effects on motivation and attention, heightened anxiety can negatively affect willingness to participate, cognitive function and flexibility [12, 15]. These findings correlate with the sense of sub-optimal performance described by participants in our study, which was associated with the level of stress they were experiencing. Anxiety was further compounded by fear of being “judged” and subsequent reputational damage in relation to their perceived “poor” performance.

Our participants reported that repeated clinical simulation exposure alleviated anxiety, supporting similar findings in previous studies in both medical students and nurses [16, 17]. The benefits of repeated and regular high-quality simulation are known to reduce anxiety as well as increase confidence and sense of clinical competence [16]. There is a lack of evidence-based research into the optimal frequency and duration of SBE activities. The barriers to implementation of SBE which limit participant exposure are well publicised [18]. It is less well recognised that excessive simulation exposure can be overwhelming for participants and will therefore have a negative influence on engagement in learning [19].

Facilitator humility and expressions of vulnerability were appreciated by participants in our study and helped address participant reluctance based on previous negative experiences. An acknowledgement that educators are also learning and developing their practice was highly valued. The idea of facilitators as co-learners is not new, it has been proposed that this more fraternal approach may be most productive where the learning objectives are behavioural [20]. Vulnerability in corporate leadership has been widely recognised as a positive attribute in recent years. It is argued that it is beneficial in building trust, fostering innovation and elevating team performance [21]. Expressions of vulnerability by medical educators may be beneficial in role-modelling reflective practice and resilience [22]. Nevertheless, the tension between maintaining credibility as an educator and exposing vulnerability has been acknowledged [23].

A lack of clarity regarding the structure and purpose of the simulation activity resulted in increased fear and avoidance behaviour amongst participants in our study. This supports the work of Kolbe [24] who states that “Instructors need to create a setting in which trainees are not left guessing about expectations or the instructor’s point of view” *(p 90)*. Alleviating anxiety through creating transparency within educational activities is a core part of promoting psychological safety. When teams feel psychologically safe, they have a shared belief that they can take interpersonal risks, such as speaking up, asking questions and sharing ideas [25]. Psychological safety is therefore fundamental in optimising learner engagement. The importance of pre-simulation activities as a method of establishing psychological safety is well recognised [26]. Research suggests that simulation educators see pre-briefing as essential groundwork in establishing a non-threatening atmosphere and enhancing the success of the subsequent debrief conversation [27]. Our study found that statements intended to build psychological safety were labelled in quite superficial terms by participants, suggesting a paucity of belief in their sentiment and value. Our findings support those of Turner et al. [28] who uncovered discrepancies in the perceptions of facilitators and learners regarding what constitutes psychological safe environment within simulation. Further investigation into this disconnect between educator and learner is warranted given the significance of achieving a psychologically safe environment in SBE.

The pre-existing relationship between participant and facilitator influences engagement and communication in simulation events [28]. It was clear from our results that facilitator integrity is crucial in building a safe and engaging learning environment, indicating that facilitators need to be cognisant of their words, actions and behaviours during all interactions with learners, both within and outside the simulation environment.

Defensive behaviours which impacted both individual and team engagement were mentioned by a number of participants in our study. Self-protective behaviours are well recognised within medical teams and are known to hinder learning-behaviours [29]. Group-protective behaviours in simulation teams are more complex and less well documented in the literature. Experienced simulation educators will recognise the practice of simulation participants gathering around a member who they perceived to be isolated or unfairly singled-out in the debrief. We have termed this phenomenon “group shielding”. We found that this defensive practice creates an “us and them” mind-set between the facilitator and the participants. The importance of a facilitator in debriefing conversations is well recognised [30]; therefore, anything which negatively influences interaction between facilitator and participant group is likely to limit the educational value of the debrief. Division between facilitator and participant group may also have an undesirable impact on the reputation of the facilitator or on SBE as a whole, resulting in future disengagement and avoidance behaviours. This study has highlighted the important yet under-recognised phenomenon of group shielding in simulation debriefing. It is hypothesised that the impact of this practice will vary depending on learning objectives and the perception of the debriefer’s role. Further research into this phenomenon would enrich current understanding of healthcare team dynamics.

## Limitations

This study was subject to several limitations. All healthcare professionals who participated in SBE within the hospital were eligible for the study; however, only doctors and nurses volunteered to take part in the interviews. The experience of doctors and nurses may not be representative of the experience of all healthcare professionals who participate in simulation. This study is based at a single hospital site in a specific cultural setting. It is well recognised that culture has a significant influence on team-based work [31]; therefore, similar research in diverse countries or ethnic group settings may provide beneficial insights into social nuances which could inform local simulation design and practice. This study used purposive sampling to recruit individuals who self-identified as reluctant simulation participants. Enrolment may have been limited by the inherent reluctance of the sample population; participants with an extreme aversion to simulation education may have been reluctant to participate in our study, which may have affected our results. Furthermore, while we attempted to reduce courtesy response bias by utilising an interviewer who had little contact with participants in simulation education contexts, the research was nevertheless carried out by the members of the hospital simulation team; thus, we cannot completely rule out bias of this nature.

## Conclusion

This study, exploring the perspective of reluctant simulation participants, offers new insights regarding engagement in SBE amongst an under-represented group in the current literature. We have found that regular simulation activities with transparent learning objectives in which facilitators demonstrate vulnerability and adopt a co-learner attitude act to reduce participant anxiety. Emergent defensive behaviours, particularly “group shielding”, interfere with collective engagement and should be both recognised and addressed by facilitators to maximise the educational value of simulation. Finally, there are potential discrepancies in the perceptions of facilitators and learners regarding what constitutes psychologically safe education environments. A collaborative and iterative approach to SBE programme design may act to improve psychological safety for reluctant participants. Based on our findings, we propose the development of an educator tool kit, to inform SBE design and practice in order to promote the engagement of reluctant simulation participants.

## Data Availability

The transcript dataset is available in de-identified format from the corresponding author upon reasonable request. The interview audio recordings have been deleted.

## Abbreviations

DSSP: Douglas Starship Simulation Programme
SBE: Simulation-based education
The Basic Assumption™: *“We believe that everyone participating in simulation activities is intelligent, capable, cares about doing their best and wants to improve”*. (© 2004–2024 Center for Medical Simulation, Boston, Massachusetts, USA. All Rights Reserved. Used with permission.)
AHREC: Auckland Health Research Ethics Committee
